# Diversity and composition of bacterial endophytes among plant parts of *Panax notoginseng*

**DOI:** 10.1186/s13020-018-0198-5

**Published:** 2018-08-14

**Authors:** Linlin Dong, Ruiyang Cheng, Lina Xiao, Fugang Wei, Guangfei Wei, Jiang Xu, Yong Wang, Xiaotong Guo, Zhongjian Chen, Shilin Chen

**Affiliations:** 1 Institute of Chinese Materia Medica, China Academy of Chinese Medical Sciences, Beijing, 100700 China; 2 Wenshan Miaoxaing Notoginseng Technology, Co., Ltd., Wenshan, 663000 China; 3 Institute of Sanqi Research, Wenshan University, Wenshan, 663000 China; 4 College of Agriculture, Ludong University, Yantai, 264025 China

**Keywords:** *Panax notoginseng*, Endophytes, Plant parts, High-throughput sequencing, 16S ribosomal RNA

## Abstract

**Background:**

Bacterial endophytes are widespread inhabitants inside plant tissues that play crucial roles in plant growth and biotransformation. This study aimed to offer information for the exploitation of endophytes by analyzing the bacterial endophytes in different parts of *Panax notoginseng*.

**Methods:**

We used high-throughput sequencing methods to analyze the diversity and composition of bacterial endophytes from different parts of *P. notoginseng*.

**Results:**

A total of 174,761 classified sequences were obtained from the analysis of 16S ribosomal RNA in different parts of *P. notoginseng.* Its fibril displayed the highest diversity of bacterial endophytes. Principal coordinate analysis revealed that the compositions of the bacterial endophytes from aboveground parts (flower, leaf, and stem) differed from that of underground parts (root and fibril). The abundances of *Conexibacter*, *Gemmatimonas*, *Holophaga*, *Luteolibacter*, *Methylophilus*, *Prosthecobacter*, and *Solirubrobacter* were significantly higher in the aboveground parts than in the underground parts, whereas the abundances of *Bradyrhizobium*, *Novosphingobium*, *Phenylobacterium*, *Sphingobium*, and *Steroidobacter* were markedly lower in the aboveground parts.

**Conclusions:**

Our results elucidated the comprehensive diversity and composition profiles of bacterial endophytes in different parts of 3-year-old *P. notoginseng.* Our data offered pivotal information to clarify the role of endophytes in the production of *P. notoginseng* and its important metabolites.

**Electronic supplementary material:**

The online version of this article (10.1186/s13020-018-0198-5) contains supplementary material, which is available to authorized users.

## Background

*Panax notoginseng* is renowned for its remarkable antihypertensive, antithrombotic, anti-atherosclerotic, and neuroprotective bioactivities, making it one of the most valuable ingredients in staple household medicines [1–3]. Protopanaxadiol and protopanaxatriol saponins are the main active compounds detected in the different parts of *P. notoginseng* [4, 5]. *P. notoginseng* is a perennial plant cultivated in fixed plots, and continuous cropping leads to decreased productivity, reduced tuber quality, and even seedling death [6, 7]. Approximately 8–10 years of crop rotation are necessary for improving the soil conditions for planted *P. notoginseng* [8]. *P. notoginseng* has a narrow ecological range, and its production mainly occurs in Wenshan, Yunnan Province, where the climatic and soil conditions are optimal for its cultivation. Nevertheless, arable soils available for *P. notoginseng* cultivation are becoming scarce.

Endophytic bacteria localized inside plant tissues have shown no negative effect on their host plants [9]. Bacteria inhabit different plant tissues, including rhizosphere, root, leaf, and stem [10]. Bacterial endophytes play key roles in improving plant growth, increasing tolerance against biotic factors, and producing secondary metabolites [11, 12]. Song et al. reported that endophytic *Bacillus altitudinis* isolated from *Panax ginseng* enhanced ginsenoside accumulation [13]. Gao et al. reported that *Paenibacillus polymyxa* isolated from *P. ginseng* leaves improved plant growth, increased ginsenoside concentration, and reduced morbidity [14]. Endophytes stimulated secondary metabolites and enhanced plant growth. Numerous works highlighted that the composition of the bacterial endophytes were influenced by plant species, parts, and growth stage [11, 15]. Analysis of the diversity and composition of endophytes in plant parts could provide valuable resources for plant growth promotion and biotransformation [16]. Although the diversity and composition of root endogenous bacteria in *P. notoginseng* have been described [17], limited information is available on the endophytic community in different parts of *P. notoginseng*. Thus, the diversity and composition of bacterial endophytes must be investigated to exploit the agronomical and metabolic potential of *P. notoginseng*.

Cultivation-independent methods can facilitate a rapid analysis of vast samples and provide reliable information on the diversity and composition of endophytic bacteria [18]. Many studies have explored rhizosphere and plant-associated bacterial communities by using high-throughput sequencing analysis [19–21]. Metagenetic methods for analyzing endophyte communities would provide deeper insight into the diversity and composition of bacterial endophytes, thereby leading to the potential discovery of new endophytes [22, 23]. Checcucci et al. reported the high taxonomic diversity of bacterial endophytes in the leaves of *Thymus* spp. by using 16S rRNA gene metagenomic sequencing [24]. A total of 29 culturable bacterial endophytes have been identified in the tissues of *Aloe vera* and characterized to 13 genera [25]. Nevertheless, culture-dependent biodiversity studies on endophytic bacterial communities remain scarce [19]. Pyrosequencing could detect low-abundance bacteria in leaf salad vegetables that could not be identified by culture-dependent methods [26]. Additional, high-throughput sequencing also used in the analysis of soil microbial communities, and this method effectively revealed the changes in diversity of soil microbial communities in soils during the cultivation of *Panax* plants [21, 27, 28]. In the present study, high-throughput sequencing analysis of 16S ribosomal RNA (rRNA) genes was conducted to describe the diversity and composition of associations among different parts of *P. notoginseng*. The results clarified the tissue-wise diversity of bacterial endophytes in the samples collected from *P. notoginseng* as well as expanded the knowledge on plant–microbe relationships and the potential properties for plant growth promotion and biotransformation.

## Methods

### Processing of samples

Three-year-old *P. notoginseng* plants were collected from Wenshan, Yunnan Province, China, which is the main production area of *P. notoginseng*. These plant samples were used to analyze the bacterial endophytes in different parts of *P. notoginseng*. Six plants were randomly gathered from one plantation and served as one sample in our test sites of Wenshan Miaoxiang Notoginseng Technology, Co., Ltd. in August. There were three replicates from three plantations. The flowers (Fl), leaves (Le), stems (St), roots (Ro), and fibers (Fi) of all samples were separated, washed with running tap water, and rinsed thrice with distilled water. A single sample consisted of 1 g of each part from six plants as one sample. To sterilize the surface of the plant parts, the samples from each part were successively immersed in 70% ethanol for 5 min, 2.5% sodium hypochlorite for 1–2 min, and 70% ethanol for 1 min, and then rinsed five times with sterile Millipore water. The last portion of the washing water was inoculated in Luria–Bertani agar at 37 °C for 24 h to validate sterilization efficiency. A total of 15 samples were stored at − 80 °C until DNA extraction.

### DNA extraction, polymerase chain reaction (PCR) amplification, and sequence processing

The total genomic DNA was extracted from all plant parts by using the MOBIO PowerSoil^®^ Kit (MOBIO Laboratories, Inc., Carlsbad, CA, USA) in accordance with the manufacturer’s instructions. The DNA quality of each sample was confirmed by utilizing a NanoDrop spectrophotometer (Thermo Fisher Scientific, Model 2000, MA, USA) and stored at − 20 °C for further PCR amplification. Bacterial 16S rRNA V1 hypervariable region genes were amplified by using the universal primers 27F/338R [29]. The forward and reverse primers contained an 8 bp barcode (Additional file 1: Table S1). PCRs were performed as described by Dong et al. with slight modifications [21]. The reaction systems were denatured at 94 °C for 3 min and then amplified for 25 cycles at 94 °C for 45 s, 55 °C for 30 s, and 72 °C for 60 s. A final extension of 10 min was added at the end of the program. Negative controls (no templates) were included to check DNA contamination of the primer or the sample. PCR products from each sample were separated with 1% agarose gel, purified with a MinElute Gel Extraction Kit (Qiagen, Valencia, CA, USA), and quantified with a Quant-iT PicoGreen dsDNA Assay Kit (Invitrogen, Carlsbad, CA, USA). The amplicons were pooled in equimolar ratios. The amplicon libraries were paired-end sequenced (2 × 250) by using an Illumina MiSeq platform in accordance with the manufacturer’s protocol.

### Data analysis

The data were processed by utilizing the QIIME pipeline [30]. Bacterial sequences were trimmed and assigned to each sample based on their barcodes. Sequences were binned into operational taxonomic units (OTUs) at 97% similarity level by using UPARSE (version 7.1 http://drive5.com/uparse/). Chimeric sequences were identified and removed by using UCHIME. The phylogenetic affiliation of each 16S rRNA gene sequence was analyzed by using a RDP Classifier (http://rdp.cme.msu.edu/) against the Silva (SSU123) 16S rRNA database at a confidence threshold of 70% [31]. Rarefaction analysis based on Mothur v.1.21.1 was conducted to reveal the diversity indices, including Chao 1 and Shannon [32]. Principal coordinate analysis (PCoA) was performed to examine dissimilarities in the community composition among samples on the basis of Bray–Curtis distance metrics [33]. Statistical analyses were performed by using the *R* package [34].

### Statistical analyses

SPSS version 16.0 was used for the statistical analyses (SPSS Inc., Chicago, IL, USA). The data were presented as mean ± SD of *n *= 3. No adjustments were implemented for multiple comparisons. The parameters were obtained for all treatment replicates and subjected one-way ANOVA.

The Minimum Standards of Reporting Checklist contains details of the details of the experimental design, and statistics, and resources used in this study (Additional file 2).

## Results

### Alpha diversity of bacterial endophytes among *P. notoginseng* parts

**Fig. 1:**
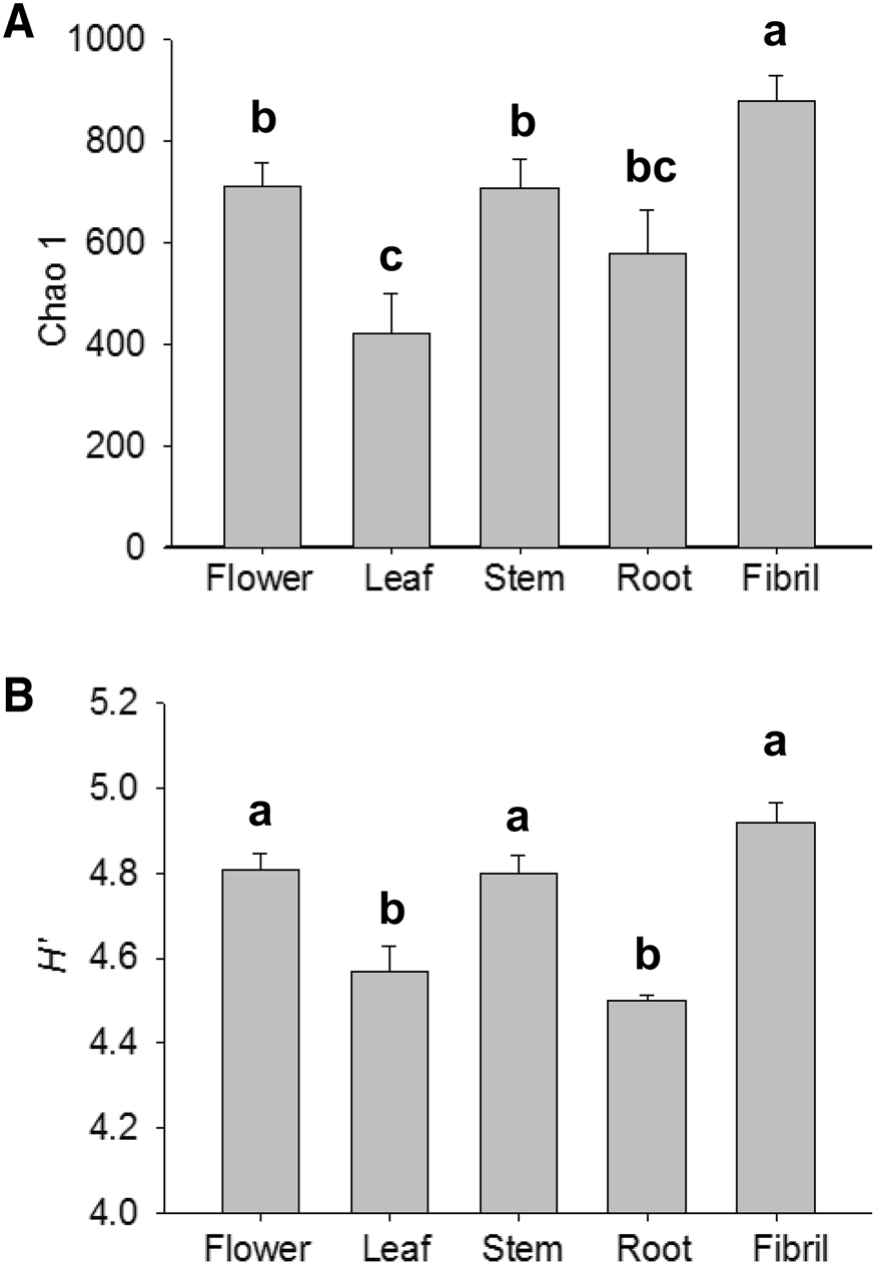
Changes of Chao 1 (**A**) and Shannon indices (**B**) for the 16S rRNA gene sequences from samples of *P. notoginseng* parts. The value of each bar represents the mean ± SD of *n *= 3. Different letters indicated significant difference at the 0.05 level

A total of 174,761 reads, with an average of 11,650 sequences per sample, were obtained from 15 samples through high-throughput sequencing analyses of 16S rRNA gene sequences (Table 1). A total of 10,351 OTUs, which ranged from 254 to 964, were found in all sequences. Alpha diversity indices (Chao 1 and Shannon) presented differences among the plant parts of *P. notoginseng* (Fig. 1). Chao 1 indicated a high number of species in the fibril samples and a low number of species in the leaf and root samples (Fig. 1A). *H’* revealed that the fibril samples had the highest diversity, whereas the root sample had the lowest diversity (Fig. 1B). The flower and stem displayed similar diversity levels.

**Table 1:** Bacterial numbers of sequences and OUTs in each sample

Samples	Sequences	OTUs
Flower-1	10,784	877
Flower-2	6818	657
Flower-3	23,711	908
Leaf-1	666	254
Leaf-2	7279	678
Leaf-3	806	256
Stem-1	22,538	849
Stem-2	2683	533
Stem-3	18,277	817
Root-1	4402	388
Root-2	12,424	726
Root-3	5944	676
Fibril-1	18,206	922
Fibril-2	12,035	846
Fibril-3	28,188	964

-1, -2, and -3 present three replicates of each *P. notoginseng* part

### Beta diversity of bacterial endophytes among *P. notoginseng* parts

**Fig. 2:**
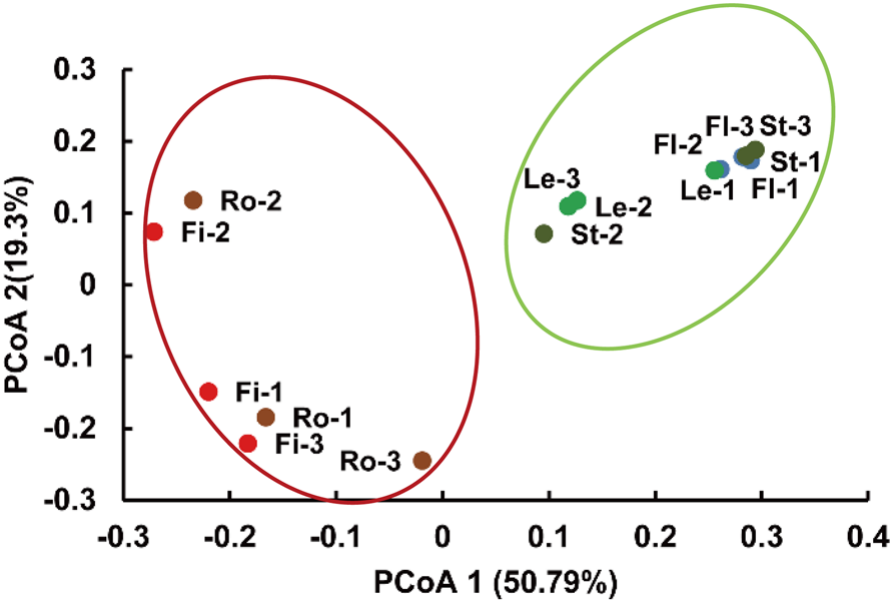
PCoA ordination plots of bacterial endophytes based on the Bray–Curtis distances of the classified 16S rRNA gene sequences. Fl, Le, St, Ro, and Fi denote the flower, leaf, stem, root, and fibril samples, respectively. -1, -2, and -3 indicate the first, second, and third replicates of each *P. notoginseng* part, respectively

The Bray–Curtis dissimilarity matrix was calculated to differentiate the bacterial communities among *P. notoginseng* parts (Fig. 2). PCoA ordination showed a strong clustering of the bacterial communities of underground (root and fibril) and aboveground parts (flower, leaf, and stem). The first and second principal components explained 50.79 and 19.3% of the total variation. PCoA indicated that the samples collected from the aboveground parts had similar profiles, whereas the samples from the underground parts were clustered together at opposite sides of the plots of the aboveground parts. Bacterial diversity in the aboveground parts differed from that in the underground parts.

### Composition of bacterial endophytes among *P. notoginseng* parts

**Fig. 3:**
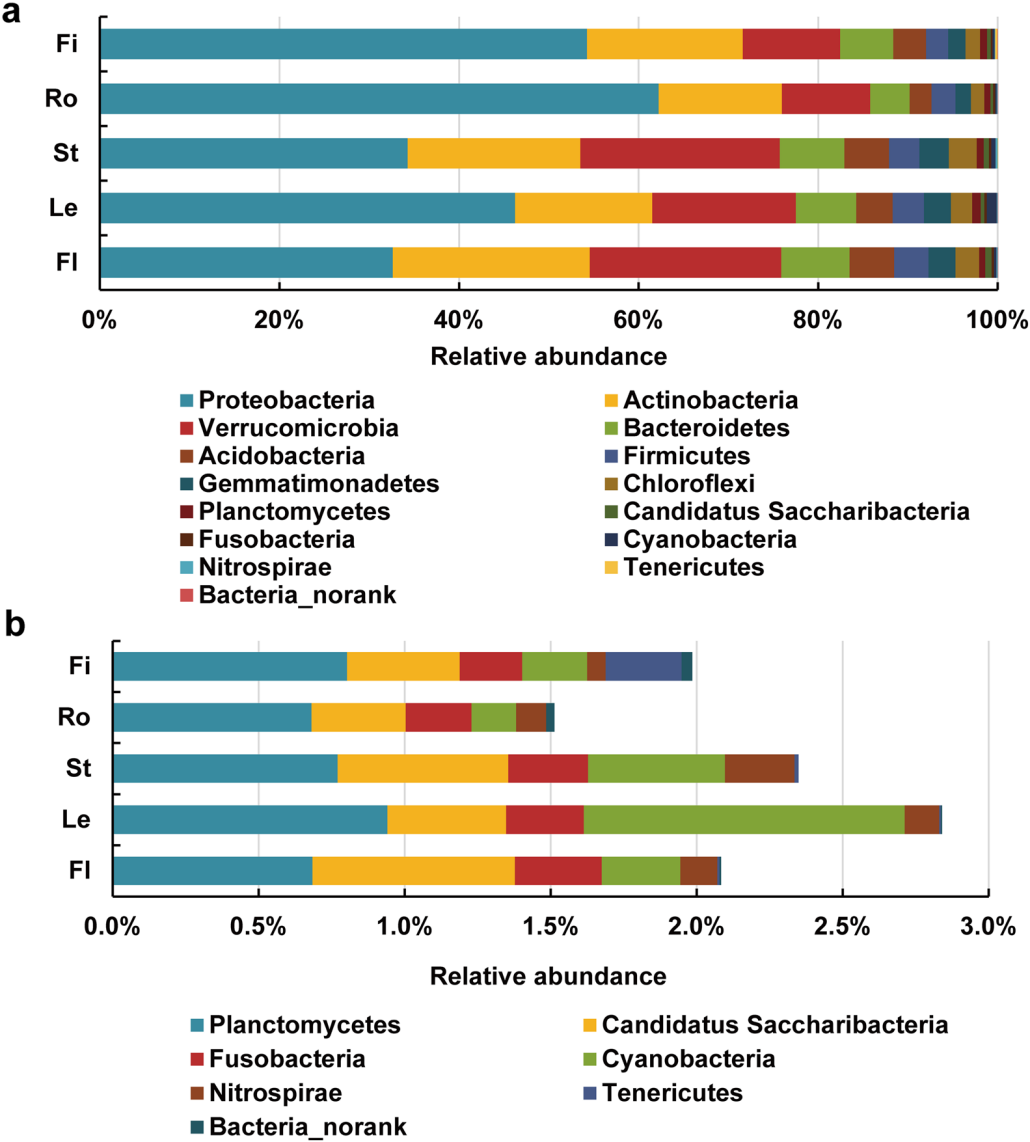
Relative abundances of the bacterial community at the phylum level. **a** All bacterial endophytes at the phylum level. **b** Bacterial endophytes with low relative abundance (< 1%) at the phylum level. Fl, Le, St, Ro, and Fi refer to the flower, leaf, stem, root, and fibril samples, respectively. The value of each bar represents the mean of *n *= 3

The reads from the 16S rRNA amplicon sequences that were generated from all samples mostly belonged to the 14 phyla (Fig. 3). The relative abundances of Proteobacteria, Actinobacteria, Verrucomicrobia, Bacteroidetes, Acidobacteria, Firmicutes, Gemmatimonadetes, and Chloroflexi reached 97.9, 97.2, 97.6, 98.2, and 97.7% in the samples from the flower, leaf, stem, root, and fibril, respectively (Fig. 3a). The relative abundance of Proteobacteria in the underground parts was significantly higher than in the aboveground parts. The abundance of Verrucomicrobia in the aboveground parts was markedly higher than in the underground parts. The relative abundances (< 1%) of Candidatus Saccharibacteria, Fusobacteria, Cyanobacteria, and Nitrospirae in the aboveground parts were higher than those in the underground parts (Fig. 3b). The relative abundance of bacterial endophytes varied and depended on the *P. notoginseng* parts at the phylum level.

**Fig. 4:**
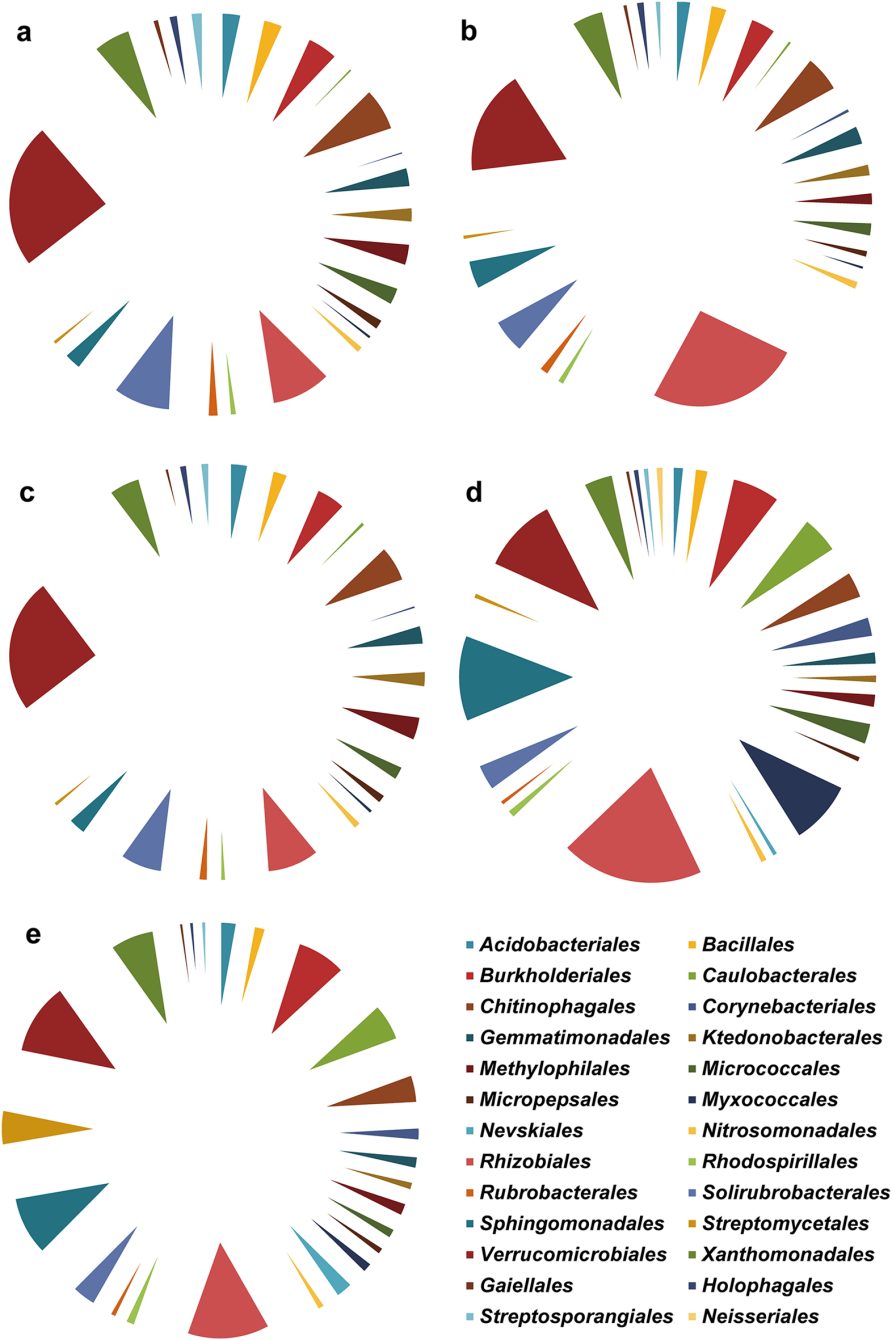
Relative abundances of bacterial community (> 1%) at the order level. **a**–**e** Abundances of the bacterial endophytes from Fl, Le, St, Ro, and Fi. The value of each bar represents the mean of *n *= 3

The relative abundances of the bacterial endophytes in the aboveground and underground parts showed considerable variation at the order level (Fig. 4 and Additional file 1: Table S2). The relative abundances of Bacillales, Chitinophagales, Gemmatimonadales, Solirubrobacterales, and Verrucomicrobiales in the aboveground parts were markedly higher than in the underground parts (*P *< 0.05). The abundances of Burkholderiales, Caulobacterales, Corynebacteriales, Myxococcales, and Sphingomonadales in the underground parts were significantly higher than those in the aboveground parts (*P *< 0.05). The bacterial endophytes in the samples from the aboveground parts with higher abundance at the order level were Chitinophagales, Rhizobiales, Solirubrobacterales, and Verrucomicrobiales. The bacterial endophytes in the samples from the underground parts with higher abundance at the order level were Burkholderiales, Rhizobiales, Sphingomonadales, and Verrucomicrobiales.

**Fig. 5:**
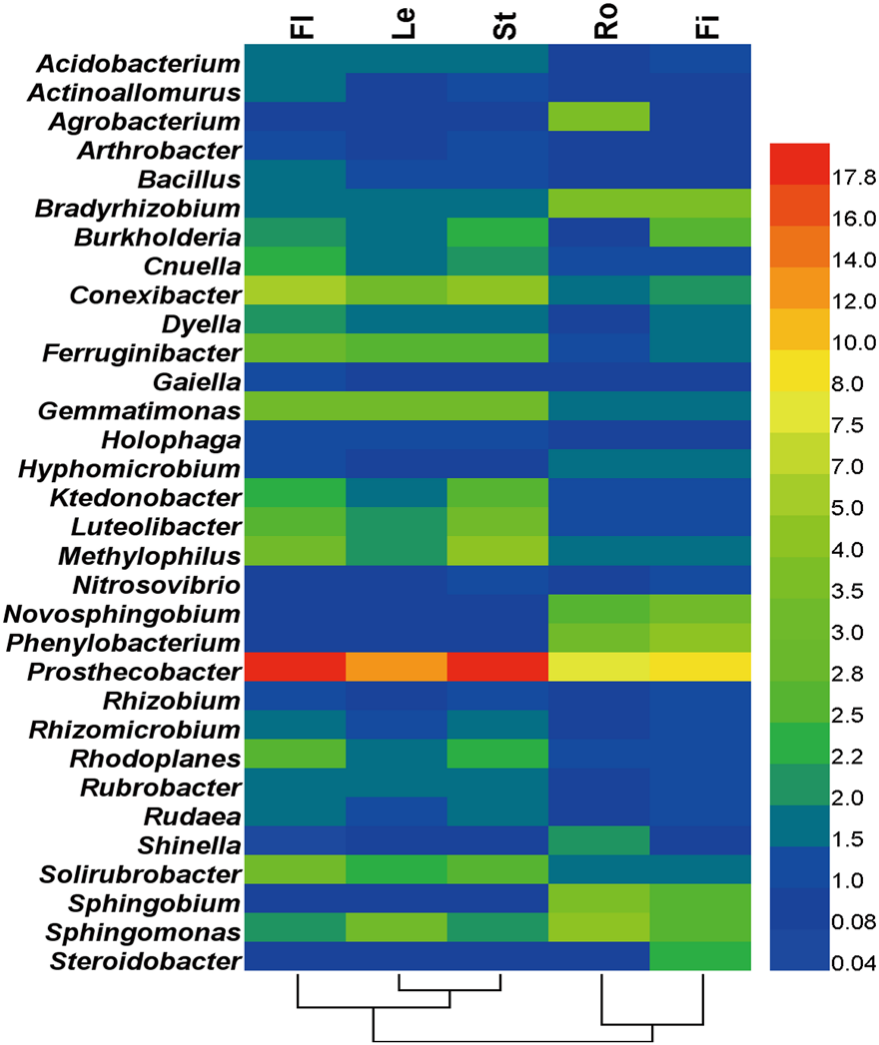
Relative abundances of the main bacterial community at the genus level. Bacterial endophytes with relative abundance (> 1%) in one treatment are shown. Fl, Le, St, Ro, and Fi refer to the flower, leaf, stem, root, and fibril samples, respectively. The value of each bar represents the mean of *n *= 3

Heat map analysis of the relative abundances of bacterial endophytes at the genus level showed variations in the samples from the aboveground and underground parts (Fig. 5). The relative abundances of *Conexibacter*, *Gemmatimonas*, *Holophaga*, *Luteolibacter*, *Methylophilus*, *Prosthecobacter*, and *Solirubrobacter* in the aboveground parts were significantly higher than those in the underground parts (*P *< 0.05). The abundances of *Bradyrhizobium*, *Novosphingobium*, *Phenylobacterium*, *Sphingobium*, and *Steroidobacter* in the aboveground parts were markedly lower than those in the underground parts (*P *< 0.05). Among all samples, *Prosthecobacter* had the highest abundance at the genus level. The low abundance (< 1.0%) of bacterial endophytes was related to the samples from *P. notoginseng* parts at the genus level (Additional file 1: Table S3). The relative abundances of *Agrobacterium*, *Sphingobium* and *Shinella* in the root samples were significantly higher than in the fibril samples (*P *< 0.05). By contrast, the abundances of *Burkholderia* and *Steroidobacter* in the root samples were markedly lower than in the fibril samples (*P *< 0.05).

## Discussion

In this study, the diversity of bacterial endophytes was associated with the different parts of *P. notoginseng*. Chao 1 and *H*’ indices indicated that the fibril samples had the highest diversity among all of the samples from the different parts. Chao 1 revealed that *Stellera chamaejasme* L. displayed an increasing trend in species richness from the root samples to the stem and leaf samples [19]. The OTUs of the bacterial endophytes were randomly distributed among plant species and organs, and Chao 1 also revealed that the diversity of *Santiria apiculate* and *Rothmannia macrophylla* in the root samples was higher than that in the leaf samples [35]. PCoA showed that samples from the aboveground parts were distinguishable from those from the underground parts. Principal component analysis (PCA) revealed that the leaf and stem samples of *S. chamaejasme* L. were clustered together and were different from the plots for the root [19], and that the stem and leaf samples of poplar trees were distinguishable from the root samples [10]. The bacterial endophytes from the fibril had the highest diversity.

In this study, Proteobacteria, Actinobacteria, Verrucomicrobia, Bacteroidetes, Acidobacteria, and Firmicutes were the main bacterial communities in *P. notoginsen*g plants. A previous study detected Proteobacteria, Actinobacteria, Bacteroidetes, and Acidobacteria in the *P. notoginseng* root [17]. Proteobacteria, Actinobacteria, Bacteroidetes, and Firmicutes were found in *P. ginseng* roots by using a culture-dependent method [16]. More than 300 endophytic actinobacteria and bacteria belonging to *Rhodococcus*, *Brevibacterium*, *Nocardioides*, *Streptomyces*, *Microbacterium*, *Nocardiopsis*, *Brachybacterium*, *Tsukamurella*, *Arthrobacter*, and *Pseudonocardia* were isolated from different tissues of *Dracaena cochinchinensis* L. [12]. The plant species influenced the selection of endophytes. The plant parts of *P. notoginseng* represented the ecological niches for bacterial endophytes.

The composition of bacterial endophytes from the aboveground parts varied from that of the underground parts. The composition of bacterial endophytes was associated with the plant compartments [10]. The relative abundances of the bacterial endophytes in all samples showed considerable variations at the phylum and genus levels [19]. The relative abundances of the bacterial endophytes, including *Conexibacter*, *Gemmatimonas*, *Holophaga*, *Luteolibacter*, *Methylophilus*, *Prosthecobacter*, *Solirubrobacter*, *Bradyrhizobium*, *Novosphingobium*, *Phenylobacterium*, *Sphingobium*, and *Steroidobacter*, in the aboveground and underground parts differed significantly. Evident strains are *Gemmatimonas, Bradyrhizobium*, *Novosphingobium*, and *Sphingobium*, which can solubilize insoluble elements, induce plant stress resistance or produce antifungal antibiotics [36–39]. Endophytic *Bacillus altitudinis* served as elicitors of biomass and ginsenoside production [13]. Bacterial endophytes from *Zea* displayed anti-fungal activity against two fungal pathogens [40]. Li et al. have reported that the domain genera included *Rhizobium*, *Sulfurospirillum*, *Uliginosibacterium*, *Pseudomonas*, *Aeromonas* and *Bacteroides*, all of which could fix nitrogen and improve plant growth [41]. The fungal endophytes communities in *Monarda citriodora* expressed anticancer and antimicrobial activities [42]. In view of the roles played by endophytes in plant growth and biotransformation, our findings contribute to the expansion of endophyte use in the production of *P. notoginseng* and its important metabolites. The information on the differences of endophytes in the aboveground and underground parts can serve as basis for the selection of functional bacteria. Importantly, higher saponins contents were detected in harvest 3-year-old *P. notoginseng* plants [43, 44]. Endophytes increased ginsenoside concentration and reduced morbidity [13, 14]. Thus, 3-year-old *P. notoginseng* plants served as the proper samples to analyze the endophytes. Additionally, the diversity of bacterial endophytes showed richness than fungal endophytes or exogenous bacteria in hour study (data not shown), and we focused on bacterial endophytes in different parts of *P. notoginseng*.

## Conclusions

The diversity and composition of bacterial endophytes were associated with different plant parts of *P. notoginseng*, and bacterial endophytes from aboveground parts (flower, leaf, and stem) were distinguished from those from underground parts (root and fibril). Our results described the profiles of bacterial endophytes in *P. notoginseng* parts and provided insight into the exploitation of endophytes in the production of *P. notoginseng* and its important metabolites.

## Additional files

**Additional file 1: Table S1.** Barcodes used to tag the PCR products. **Table S2.** Relative abundance (<1.0%) of the bacterial taxa at the order level. **Table S3.** Relative abundance (<1.0%) of the bacterial taxa at the genus level.

**Additional file 2.** Minimum Standards of Reporting Checklist.

## Abbreviations

Fl: flower
Le: leaf
St: stem
Ro: root
Fi: fibril
rRNA: ribosomal RNA
PCR: polymerase chain reaction
PCoA: principal coordinates analysis
RDP: ribosomal database project
OTUs: operational taxonomic units
*H*’: Shannon index
