# Supplementary material for: Diversity and composition of bacterial endophytes among plant parts of Panax notoginseng
Source: Chin Med. 2018 Aug 14;13:41. doi: 10.1186/s13020-018-0198-5 (PMC6092820; doi:10.1186/s13020-018-0198-5)
Supplement: Supplementary file 1 — Additional file 1: Table S1. Barcodes used to tag the PCR products. Table S2. Relative abundance (<1.0%) of the bacterial taxa at the order level. Table S3. Relative abundance (<1.0%) of the bacterial taxa at the genus level. [file 13020_2018_198_MOESM1_ESM.docx]

Table S1. Barcodes used to tag the PCR products.

| Samples | Barcodes |
| --- | --- |
| Flower-1 | CTTAGATA |
| Flower-2 | CGCTGTAA |
| Flower-3 | GACATTCA |
| Leaf-1 | GTAACTTA |
| Leaf-2 | GAGGGTTA |
| Leaf-3 | GGCCCAAT |
| Stem-1 | CTAAGTTA |
| Stem-2 | TTGTGCTA |
| Stem-3 | TAACGCTT |
| Root-1 | TGGCATTA |
| Root-2 | TCCGTCTA |
| Root-3 | ATTGCCAT |
| Fibril-1 | TCGATCAT |
| Fibril-2 | TGCTACTT |
| Fibril-3 | TAAGCAGC |

-1, -2, and -3 present three replicates of each *P. notoginseng* part.

Table S2. Relative abundance (<1.0%) of the bacterial taxa at the order level.

| OTU ID | | Fl | Le | | | St | Ro | | Fi | |
| --- | --- | --- | --- | --- | --- | --- | --- | --- | --- | --- |
| Acidimicrobiales | 0.423±0.027a | | 0.329±0.022a | 0.309±0.065a | | 0.521±0.069a | 0.358±0.078a | |  |  |
| Acidothermales | 0.019±0.013b | | 0.050±0.050a | 0.023±0.009b | | 0.006±0.001b | 0.017±0.007b | |  |  |
| Actinomycetales | 0.495±0.036a | | 0.545±0.100a | 0.588±0.052a | | 0.172±0.114b | 0.296±0.158b | |  |  |
| Alteromonadales | 0.008±0.004b | | 0.091 ±0.046b | 0.039±0.037b | | 0.383±0.044a | 0.095±0.065b | |  |  |
| Anaerolineales | 0.075±0.107a | | 0.078±0.043a | 0.108±0.020a | | 0.035±0.013a | 0.068±0.018a | |  |  |
| Bacteroidales | 0.085±0.009b | | 0.078±0.039b | 0.049±0.025b | | 0.087±0.051b | 0.590±0.051a | |  |  |
| Bdellovibrionales | 0.006±0.004a | | 0.000±0.000a | 0.002±0.002a | | 0.079±0.063a | 0.035±0.015a | |  |  |
| Caldilineales | 0.047±0.006a | | 0.009±0.009a | 0.050±0.022a | | 0.020±0.016a | 0.034±0.017a | |  |  |
| Campylobacterales | 0.021±0.011a | | 0.005±0.046a | 0.016±0.030a | | 0.006±0.001a | 0.022±0.005a | |  |  |
| Cellvibrionales | 0.006±0.027b | | 0.091±0.089b | 0.032±0.002b | | 0.495±0.091a | 0.156±0.027b | |  |  |
| Chromatiales | 0.162±0.009a | | 0.132±0.000a | 0.166±0.004a | | 0.330±0.108a | 0.345±0.078a | |  |  |
| Chroococcales | 0.013±0.051a | | 0.000±0.050a | 0.003±0.006a | | 0.024±0.015a | 0.050±0.012a | |  |  |
| Clostridiales | 0.769±0.002a | | 0.718±0.009a | 0.703±0.016a | | 0.382±0.211b | 0.511±0.160ab | |  |  |
| Cytophagales | 0.030±0.018b | | 0.009±0.073b | 0.033±0.006b | | 0.186±0.101a | 0.236±0.062a | |  |  |
| Desulfuromonadales | 0.408±0.016a | | 0.265±0.005b | 0.456±0.017a | | 0.514±0.285a | 0.631±0.087a | |  |  |
| Elusimicrobiales | 0.011±0.066a | | 0.005±0.145a | 0.011±0.040a | | 0.081±0.003a | 0.014±0.004a | |  |  |
| Enterobacterales | 0.038±0.004b | | 0.230±0.037a | 0.041±0.039b | | 0.329±0.185a | 0.064±0.037b | |  |  |
| Flavobacteriales | 0.023±0.079a | | 0.101±0.053a | 0.072±0.0.48a | | 0.152±0.114a | 0.177±0.043a | |  |  |
| Frankiales | 0.818±0.012a | | 0.384±0.134b | 0.821±0.073a | | 0.267±0.045b | 0.320±0.142b | |  |  |
| Fusobacteriales | 0.298±0.018a | | 0.266±0.131a | 0.273±0.004a | | 0.225±0.005a | 0.214 ±0.052a | |  |  |
| Geodermatophilales | 0.984±0.008a | | 0.540±0.041b | 0.928±0.032a | | 0.409±0.020b | 0.470±0.188b | |  |  |
| Holosporales | 0.021±0.033a | | 0.041±0.046a | 0.004±0.008a | | 0.008±0.002a | 0.015±0.012a | |  |  |
| Kineosporiales | 0.127±0.040a | | 0.091±0.046a | 0.101±0.032a | | 0.028±0.047a | 0.071±0.030a | |  |  |
| Lactobacillales | 0.016±0.059a | | 0.059±0.005a | 0.022±0.012a | | 0.021±0.011a | 0.007±0.001a | |  |  |
| Legionellales | 0.249±0.019a | | 0.110±0.092a | 0.235±0.105a | | 0.224±0.058a | 0.263±0.048a | |  |  |
| Methylococcales | 0.016±0.107a | | 0.000±0.018a | 0.014±0.019a | | 0.011±0.000a | 0.003±0.002a | |  |  |
| Micromonosporales | 0.190±0.025a | | 0.091±0.046a | 0.157±0.064a | | 0.120±0.084a | 0.140±0.037a | |  |  |
| Mycoplasmatales | 0.007±0.047a | | 0.005±0.001a | 0.014±0.004a | | 0.000±0.000a | 0.260±0.220a | |  |  |
| Nitrospirales | 0.127±0.100a | | 0.118±0.089a | 0.238±0.013a | | 0.103±0.015a | 0.063±0.020a | |  |  |
| Opitutales | 0.032±0.006a | | 0.018±0.107a | 0.032±0.029a | | 0.247±0.027a | 0.172±0.033a | |  |  |
| Oscillatoriales | 0.166±0.042a | | 0.046±0.036a | 0.120±0.027a | | 0.038±0.014a | 0.080±0.031a | |  |  |
| Parachlamydiales | 0.012±0.007a | | 0.000±0.000a | 0.004±0.034a | | 0.105±0.101a | 0.243±0.120a | |  |  |
| Phycisphaerales | 0.069±0.008a | | 0.091±0.188a | 0.096±0.059a | | 0.022±0.010a | 0.079±0.025a | |  |  |
| Planctomycetales | 0.614±0.033a | | 0.849±0.065a | 0.675±0.316a | | 0.659±0.027a | 0.724±0.087a | |  |  |
| Propionibacteriales | 0.757±0.071a | | 0.416±0.120a | 0.555±0.019a | | 0.290±0.065b | 0.412±0.135a | |  |  |
| Pseudomonadales | 0.354±0.039a | | 0.585±0.447a | 0.499±0.016a | | 0.713±0.152a | 0.922±0.476a | |  |  |
| Pseudonocardiales | 0.248±0.003a | | 0.092±0.100a | 0.214±0.112a | | 0.089±0.007a | 0.094±0.035a | |  |  |
| Rhodobacterales | 0.070±0.040b | | 0.069±0.120b | 0.060±0.001b | | 0.271±0.035a | 0.108±0.019a | |  |  |
| Rhodocyclales | 0.172±0.059b | | 0.105±0.060b | 0.240±0.021b | | 0.653±0.070a | 0.488±0.188a | |  |  |
| Solibacterales | 0.559±0.019a | | 0.325±0.044ab | 0.462±0.031a | | 0.253±0.196b | 0.326±0.068ab | |  |  |
| Sphaerobacterales | 0.198±0.107a | | 0.252±0.107a | 0.177±0.006a | | 0.159±0.007a | 0.122±0.051a | |  |  |
| Sphingobacteriales | 0.864±0.025a | | 0.727±0.044a | 0.816±0.102a | | 0.400±0.048b | 0.605±0.096ab | |  |  |
| Spirulinales | 0.069±0.047b | | 0.990±0.100a | 0.338±0.312ab | | 0.075±0.006b | 0.066±0.030b | |  |  |
| Syntrophobacterales | 0.312±0.009a | | 0.449±0.065a | 0.281±0.109a | | 0.148±0.048a | 0.195±0.066a | |  |  |
| Thermoanaerobacterales | 0.025±0.006b | | 0.068±0.044b | 0.032±0.016b | | 0.519±0.274a | 0.082±0.009b | |  |  |
| Thermoleophilales | 0.671±0.021a | | 0.338±0.107a | 0.521±0.112a | | 0.272±0.210a | 0.296±0.123a | |  |  |

Fl, Le, St, Ro and Fi represented samples from flower, leaf, stem, root and fibril, respectively. Different letters denote a significant difference between plant parts at the level of 0.05. The data represent the mean ± SD of *n*=3.

Table S3. Relative abundance (<1.0%) of the bacterial taxa at the genus level.

| OTU ID | Fl | Le | St | Ro | Fi |
| --- | --- | --- | --- | --- | --- |
| *Acidimicrobium* | 0.059±0.006a | 0.096±0.006a | 0.028±0.002a | 0.032±0.007a | 0.040±0.007a |
| *Acidisphaera* | 0.079±0.012a | 0.050±0.008a | 0.122±0.032a | 0.044±0.009a | 0.044±0.005a |
| *Aciditerrimonas* | 0.137±0.008a | 0.110±0.007a | 0.111±0.014a | 0.107±0.008a | 0.119±0.007a |
| *Actinomadura* | 0.334±0.142a | 0.211±0.103a | 0.341±0.015a | 0.155±0.012a | 0.145±0.021a |
| *Actinoplanes* | 0.099±0.021a | 0.073±0.008a | 0.076±0.031a | 0.058±0.009a | 0.077±0.032a |
| *Aeromicrobium* | 0.063±0.031a | 0.051±0.004a | 0.056±0.006a | 0.024±0.032a | 0.029±0.014a |
| *Aliidongia* | 0.168±0.009a | 0.247±0.122a | 0.153±0.012a | 0.070±0.009b | 0.086±0.007b |
| *Amnibacterium* | 0.137±0.008a | 0.110±0.009a | 0.104±0.056a | 0.031±0.005a | 0.089±0.009a |
| *Anaeromyxobacter* | 0.165±0.011b | 0.133±0.054b | 0.160±0.014b | 0.567±0.132a | 0.351±0.012a |
| *Ancylobacter* | 0.221±0.016a | 0.142±0.062a | 0.127±0.036a | 0.055±0.007b | 0.099±0.007b |
| *Arachidicoccus* | 0.065±0.009a | 0.009±0.063a | 0.075±0.005a | 0.039±0.004a | 0.046±0.021a |
| *Arenimonas* | 0.057±0.007b | 0.069±0.007b | 0.092±0.006b | 0.135±0.074a | 0.281±0.019a |
| *Arvibacter* | 0.039±0.005a | 0.005±0.001a | 0.022±0.002a | 0.022±0.031a | 0.008±0.001a |
| *Asticcacaulis* | 0.065±0.008a | 0.027±0.004a | 0.080±0.005a | 0.119±0.011a | 0.168±0.012a |
| *Azoarcus* | 0.144±0.112a | 0.032±0.006a | 0.172±0.008a | 0.017±0.007a | 0.073±0.032a |
| *Azospirillum* | 0.162±0.018a | 0.220±0.042a | 0.134±0.004a | 0.153±0.007a | 0.153±0.047a |
| *Bacteroides* | 0.085±0.036b | 0.078±0.009b | 0.046±0.021b | 0.087±0.003b | 0.590±0.102a |
| *Blastococcus* | 0.984±0.036a | 0.540±0.062b | 0.928±0.144a | 0.409±0.133b | 0.470±0.312b |
| *Blastopirellula* | 0.117±0.021a | 0.155±0.021a | 0.127±0.022a | 0.100±0.005a | 0.161±0.079a |
| *Bordetella* | 0.044±0.004a | 0.083±0.021a | 0.033±0.003a | 0.027±0.008a | 0.338±0.111a |
| *Caulobacter* | 0.073±0.007b | 0.096±0.006b | 0.168±0.016b | 0.899±0.216a | 0.899±0.010a |
| *Clostridium* | 0.519±0.121a | 0.517±0.0.21a | 0.452±0.021a | 0.258±0.014b | 0.324±0.056 b |
| *Cohnella* | 0.017±0.009a | 0.005±0.003a | 0.021±0.003a | 0.008±0.001a | 0.015±0.007a |
| *Cupriavidus* | 0.951±0.012a | 0.403±0.021b | 0.954±0.122a | 0.351±0.036b | 0.462±0.062b |
| *Cystobacter* | 0.104±0.006a | 0.123±0.003a | 0.144±0.087a | 0.050±0.007a | 0.049±0.021a |
| *Dactylosporangium* | 0.042±0.002a | 0.018±0.004a | 0.031±0.021a | 0.028±0.021a | 0.029±0.007a |
| *Desulfitobacterium* | 0.100±0.001a | 0.064±0.005a | 0.116±0.005a | 0.067±0.005a | 0.077±0.011a |
| *Desulfovirga* | 0.178±0.041a | 0.297±0.007a | 0.213±0.122a | 0.096±0.007a | 0.121±0.008a |
| *Devosia* | 0.548±0.032a | 0.289±0.121b | 0.437±0.122ab | 0.747±0.110a | 0.483±0.132ab |
| *Dokdonella* | 0.323±0.054a | 0.279±0.004a | 0.427±0.010a | 0.225±0.009a | 0.361±0.122a |
| *Dongia* | 0.027±0.011b | 0.046±0.002b | 0.041±0.005b | 0.354±0.007a | 0.707±0.145a |
| *Ectothiorhodospira* | 0.011±0.007a | 0.009±0.002a | 0.004±0.001a | 0.028±0.032a | 0.024±0.009a |
| *Edaphobacter* | 0.440±0.005a | 0.179±0.026a | 0.332±0.056a | 0.144±0.011a | 0.300±0.211a |
| *Enterobacter* | 0.030±0.006b | 0.230±0.032a | 0.027±0.002b | 0.207±0.031a | 0.033±0.007b |
| *Eubacterium* | 0.018±0.005a | 0.014±0.021a | 0.024±0.004a | 0.024±0.004a | 0.024±0.004a |
| *Ferrimicrobium* | 0.193±0.061a | 0.078±0.026a | 0.134±0.021a | 0.111±0.007a | 0.085±0.002a |
| *Flaviaesturariibacter* | 0.147±0.021a | 0.046±0.031a | 0.177±0.036a | 0.064±0.004a | 0.097±0.007a |
| *Flavisolibacter* | 0.663±0.032a | 0.526±0.056a | 0.632±0.087a | 0.243±0.004a | 0.404±0.003a |
| *Flexivirga* | 0.085±0.056a | 0.068±0.078a | 0.070±0.012a | 0.055±0.012a | 0.036±0.006a |
| *Frankia* | 0.042±0.012a | 0.064±0.011a | 0.067±0.013a | 0.000±0.000a | 0.019±0.003a |
| *Frateuria* | 0.057±0.033a | 0.092±0.033a | 0.030±0.003a | 0.046±0.031a | 0.117±0.102a |
| *Fusobacterium* | 0.298±0.069a | 0.266±0.046a | 0.273±0.021a | 0.225±0.021a | 0.214±0.008a |
| *Gemmata* | 0.026±0.006a | 0.000±0.000a | 0.031±0.021a | 0.076±0.008a | 0.053±0.004a |
| *Geoalkalibacter* | 0.034±0.011a | 0.009±0.001a | 0.043±0.004a | 0.050±0.004a | 0.056±0.006a |
| *Geobacter* | 0.278±0.067a | 0.233±0.065a | 0.343±0.091a | 0.365±0.017a | 0.447±0.107a |
| *Granulicella* | 0.048±0.012a | 0.060±0.021a | 0.053±0.004a | 0.030±0.021a | 0.023±0.032a |
| *Haliangium* | 0.089±0.013a | 0.087±0.013a | 0.125±0.002a | 0.119±0.007a | 0.140±0.008a |
| *Halospirulina* | 0.069±0.021c | 0.993±0.132a | 0.338±0.006b | 0.075±0.004c | 0.066±0.002c |
| *Helicobacter* | 0.021±0.006a | 0.005±0.001a | 0.016±0.005a | 0.006±0.002a | 0.022±0.003a |
| *Herbaspirillum* | 0.246±0.047a | 0.155±0.087a | 0.275±0.032a | 0.132±0.071a | 0.167±0.018a |
| *Herminiimonas* | 0.231±0.046a | 0.105±0.018a | 0.266±0.065a | 0.095±0.002a | 0.126±0.012a |
| *Humibacter* | 0.060±0.031a | 0.050±0.016a | 0.035±0.005a | 0.050±0.007a | 0.081±0.017a |
| *Jatrophihabitans* | 0.350±0.032a | 0.192±0.032b | 0.372±0.015a | 0.136±0.007b | 0.136±0.021b |
| *Kineosporia* | 0.081±0.021a | 0.091±0.016a | 0.072±0.014a | 0.022±0.002a | 0.051±0.019a |
| *Kofleria* | 0.062±0.008a | 0.009±0.001a | 0.087±0.005a | 0.051±0.001a | 0.037±0.007a |
| *Legionella* | 0.248±0.124a | 0.110±0.003a | 0.226±0.027a | 0.193±0.074a | 0.214±0.027a |
| *Leifsonia* | 0.044±0.032b | 0.205±0.096a | 0.034±0.026b | 0.020±0.004b | 0.022±0.002b |
| *Lentzea* | 0.107±0.003a | 0.064±0.007a | 0.084±0.021a | 0.055±0.003a | 0.037±0.007a |
| *Leptothrix* | 0.093±0.006a | 0.023±0.008a | 0.073±0.007a | 0.124±0.047a | 0.218±0.136a |
| *Lysobacter* | 0.560±0.032a | 0.430±0.067a | 0.525±0.013a | 0.247±0.201a | 0.478±0.029a |
| *Massilia* | 0.363±0.0.26a | 0.504±0.069a | 0.303±0.019a | 0.162±0.018a | 0.217±0.067a |
| *Mesorhizobium* | 0.094±0.025b | 0.078±0.051b | 0.165±0.021ab | 0.789±0.213a | 0.340±0.065a |
| *Metallibacterium* | 0.299±0.102a | 0.297±0.064a | 0.320±0.018a | 0.076±0.008a | 0.179±0.065a |
| *Methylibium* | 0.016±0.0.13b | 0.005±0.001b | 0.055±0.023b | 1.278±0.026a | 0.458±0.021b |
| *Methylobacterium* | 0.030±0.005b | 0.390±0.005a | 0.139±0.087a | 0.182±0.007a | 0.218±0.036a |
| *Methylosinus* | 0.145±0.021a | 0.050±0.007b | 0.134±0.081a | 0.022±0.007b | 0.076±0.019ab |
| *Microbacterium* | 0.056±0.012b | 0.005±0.002b | 0.081±0.012b | 1.507±0.021a | 0.104±0.071b |
| *Mucilaginibacter* | 0.548±0.031a | 0.447±0.079a | 0.538±0.037a | 0.254±0.031a | 0.370±0.032a |
| *Mycobacterium* | 0.363±0.032c | 0.594±0.085c | 0.460±0.021c | 2.483±0.514a | 1.913±0.561b |
| *Myxococcus* | 0.022±0.023c | 0.338±0.102b | 0.060±0.007c | 6.759±1.321a | 0.671±0.032b |
| *Niastella* | 0.168±0.014b | 0.283±0.052b | 0.190±0.005b | 0.141±0.155b | 0.693±0.147a |
| *Nitrosospira* | 0.306±0.021a | 0.563±0.047a | 0.444±0.021a | 0.138±0.032a | 0.200±0.009a |
| *Nitrospira* | 0.127±0.029a | 0.118±0.041a | 0.238±0.069a | 0.103±0.065a | 0.063±0.007a |
| *Nocardioides* | 0.464±0.103a | 0.247±0.012a | 0.308±0.031a | 0.221±0.031a | 0.247±0.037a |
| *Pasteuria* | 0.012±0.017b | 0.092±0.031b | 0.046±0.011b | 0.429±0.023a | 0.312±0.049a |
| *Paucibacter* | 0.033±0.013b | 0.129±0.015b | 0.046±0.007b | 0.457±0.014a | 0.338±0.031a |
| *Pedobacter* | 0.209±0.106a | 0.220±0.061a | 0.175±0.012a | 0.084±0.008a | 0.147±0.037a |
| *Pedosphaera* | 0.291±0.103a | 0.210±0.014a | 0.346±0.03a1 | 0.232±0.064a | 0.318±0.012a |
| *Pelobacter* | 0.095±0.016a | 0.023±0.004a | 0.070±0.004a | 0.100±0.008a | 0.128±0.067a |
| *Pelotomaculum* | 0.012±0.008a | 0.009±0.001a | 0.024±0.009a | 0.006±0.001a | 0.009±0.006a |
| *Phycicoccus* | 0.127±0.019a | 0.032±0.002a | 0.127±0.012a | 0.048±0.003a | 0.060±0.002a |
| *Phycisphaera* | 0.069±0.021a | 0.091±0.001a | 0.096±0.003a | 0.022±0.004a | 0.079±0.009a |
| *Pirellula* | 0.020±0.003a | 0.005±0.001a | 0.025±0.008a | 0.110±0.007a | 0.124±0.032a |
| *Planctomyces* | 0.285±0.005a | 0.288±0.211a | 0.309±0.017a | 0.193±0.021a | 0.208±0.012a |
| *Porphyrobacter* | 0.109±0.009a | 0.018±0.003a | 0.089±0.021a | 0.100±0.021a | 0.073±0.032a |
| *Pseudolabrys* | 0.466±0.102a | 0.353±0.162a | 0.472±0.123a | 0.251±0.122a | 0.311±0.124a |
| *Pseudomonas* | 0.344±0.201a | 0.452±0.047a | 0.444±0.012a | 0.586±0.312a | 0.900±0.012a |
| *Pseudonocardia* | 0.070±0.007a | 0.018±0.002a | 0.056±0.008a | 0.017±0.012a | 0.022±0.006a |
| *Pseudorhodoferax* | 0.020±0.003a | 0.009±0.001a | 0.065±0.004a | 0.085±0.007a | 0.134±0.015a |
| *Pullulanibacillus* | 0.147±0.007a | 0.083±0.002a | 0.110±0.012a | 0.056±0.002a | 0.077±0.027a |
| *Ralstonia* | 0.064±0.002a | 0.119±0.032a | 0.079±0.007a | 0.061±0.012a | 0.066±0.032a |
| *Ramlibacter* | 0.140±0.008a | 0.156±0.062a | 0.120±0.004a | 0.168±0.122a | 0.252±0.122a |
| *Reyranella* | 0.219±0.201a | 0.246±0.012a | 0.236±0.006a | 0.196±0.074a | 0.178±0.072a |
| *Rhodanobacter* | 0.436±0.001a | 0.362±0.031a | 0.384±0.021a | 0.130±0.032a | 0.258±0.074a |
| *Rhodobium* | 0.085±0.005a | 0.069±0.002a | 0.106±0.007a | 0.061±0.012a | 0.048±0.032a |
| *Rhodomicrobium* | 0.107±0.009a | 0.087±0.005a | 0.151±0.051a | 0.061±0.084a | 0.104±0.007a |
| *Rhodothermus* | 0.154±0.021a | 0.119±0.007a | 0.123±0.012a | 0.088±0.001a | 0.104±0.008a |
| *Saccharibacter* | 0.145±0.054a | 0.032±0.008a | 0.106±0.071a | 0.064±0.032a | 0.088±0.012a |
| *Schumannella* | 0.260±0.103a | 0.220±0.012a | 0.222±0.003a | 0.248±0.056a | 0.195±0.031a |
| *Sediminibacterium* | 0.058±0.005a | 0.009±0.005a | 0.022±0.037a | 0.017±0.001a | 0.031±0.027 a |
| *Skermanella* | 0.046±0.002a | 0.078±0.001a | 0.022±0.005 a | 0.029±0.009 a | 0.033±0.006a |
| *Sphaerobacter* | 0.198±0.105a | 0.252±0.121a | 0.177±0.019a | 0.159±0.032a | 0.122±0.071a |
| *Sphingobacterium* | 0.107±0.003a | 0.060±0.006a | 0.103±0.006a | 0.063±0.021a | 0.088±0.046a |
| *Stella* | 0.230±0.132a | 0.206±0.005a | 0.186±0.021a | 0.080±0.014a | 0.126±0.095a |
| *Stenotrophobacter* | 0.024±0.006a | 0.000±0.000a | 0.027±0.013a | 0.057±0.003a | 0.032±0.013a |
| *Sterolibacterium* | 0.017±0.003a | 0.018±0.012a | 0.034±0.007a | 0.295±0.074a | 0.169±0.014a |
| *Syntrophus* | 0.131±0.009a | 0.152±0.049a | 0.068±0.012a | 0.032±0.012a | 0.067±0.027a |
| *Terrimicrobium* | 0.035±0.001a | 0.064±0.002a | 0.028±0.009a | 0.025±0.012a | 0.025±0.009a |
| *Terrimonas* | 0.100±0.008a | 0.050±0.003a | 0.148±0.032a | 0.594±0.312a | 0.206±0.031a |
| *Thermoflavimicrobium* | 0.918±0.213a | 0.773±0.231a | 0.853±0.075a | 0.343±0.019a | 0.486±0.024a |
| *Thermoleophilum* | 0.671±0.162a | 0.338±0.102b | 0.521±0.097a | 0.272±0.032b | 0.296±0.174b |
| *Thioalkalivibrio* | 0.115±0.005a | 0.064±0.007a | 0.109±0.012a | 0.105±0.037a | 0.195±0.036a |
| *Trichodesmium* | 0.166±0.008a | 0.046±0.003a | 0.120±0.008a | 0.038±0.002a | 0.080±0.021a |
| *Variovorax* | 0.249±0.065b | 0.369±0.162b | 0.291±0.102b | 0.841±0.169a | 0.802±0.068a |
| *Verrucomicrobium* | 0.059±0.007a | 0.009±0.001a | 0.050±0.007a | 0.085±0.007a | 0.050±0.012a |
| *Zavarzinella* | 0.145±0.005a | 0.347±0.003a | 0.142±0.071a | 0.106±0.007a | 0.130±0.007a |

Fl, Le, St, Ro and Fi represented samples from flower, leaf, stem, root and fibril, respectively. Different letters denote a significant difference between plant parts at the level of 0.05. The data represent the mean ± SD of *n*=3.
